# Editorial: Food and Its Effect on the Brain: From Physiological to Compulsive Consumption

**DOI:** 10.3389/fpsyt.2019.00209

**Published:** 2019-04-04

**Authors:** Valentina Bassareo, Carla Gambarana

**Affiliations:** ^1^Department of Biomedical Sciences, University of Cagliari, Cagliari, Italy; ^2^Department of Biomedical Sciences, National Institute of Neuroscience, Cagliari section, Cagliari, Italy; ^3^Department of Molecular and Developmental Medicine, University of Siena, Siena, Italy

**Keywords:** dopamine, homeostasis, motivation, orexin, food-addiction, junk-food, saikosaponin A, caffeine

Food is a primary stimulus essential for survival that can activate rewarding brain circuits through at least three senses: taste, smell, and sight. While the caloric content represents the primary value, perceived palatability is a major factor controlling feeding behaviors (1). Information about the pleasant experience is conveyed from the periphery to different levels of the central nervous system where several neuronal systems contribute to the complex regulation of food intake and energy homeostasis (2). This *Frontiers* Research Topic brings together contributions on central and peripheral mechanisms that control food intake, offering an up-to-date perspective on this subject and on relevant implications for human health.

A robust literature demonstrates the involvement of mesolimbic and mesocortical dopamine (DA) transmission in the responses to food consumption and food-related behaviors. In particular, DA transmission shows an adaptive response to highly palatable food consumption, preferentially in the nucleus accumbens shell (NAcS), a mesolimbic dopaminergic terminal area. The NAcS dopaminergic response undergoes rapid habituation despite greater consumption of the same food and shorter latency to eat it (3–10). This adaptive mechanism supports the hypothesized facilitatory role of NAcS DA in the establishment of associations between food rewarding properties and secondary stimuli that acquire predictive value (11, 12). Furthermore, other neuronal systems and homeostatic hormonal and nonhormonal signals are involved in the modulation of the rewarding and/or incentive properties of food, and most of them seem to converge on the mesolimbic dopaminergic system (13).

The role played in several domains of goal-directed behavior by phasic activation of DA neurons of the ventral tegmental area (VTA) and consequent phasic DA release in striatal terminal regions, and how they may be tuned by perturbations in physiological state, conveyed by energy balance and hormonal signals, has been pointed out by Hsu et al. These authors provide an exhaustive overview of central pathways involved in the complex integration of homeostatic signals and goal-directed behaviors, focusing on the role of the dopaminergic mesolimbic system. The authors highlight the influences of several hormones, such as glucagon, amylin, leptin, and ghrelin, on food intake and related motivated behaviors and how hormonal signaling modulates phasic mesolimbic dopaminergic output. Changes in physiological state can strongly influence motivated behaviors and reward-seeking, and the mesolimbic dopaminergic system may represent a hub that coordinates adaptive behavioral responses to such modifications. Finally, Hsu et al. report that the caloric content and hedonic value of a food stimulus elicit different responses in dorsal and ventral striatal areas. A relevant question to be addressed is whether hedonic and caloric values are processed through distinct dorsal versus ventral dopaminergic striatal pathways or *via* integrated pathways within these regions.

In the context of homeostatic regulation of VTA DA transmission by hormonal signals, the role of orexin A (OxA), a neuropeptide produced by neurons in the lateral hypothalamus (LH) that stimulates food intake (14), has been investigated by Lai et al. In order to examine OxA modulation of basal and feeding-activated NAcS dopaminergic transmission, this large peptide that does not cross the blood–brain barrier was intravenously administered using liposomes as innovative delivery vehicles. OxA stimulates food intake and increases DA transmission in the NAcS at baseline and in response to palatable food, and the administration of an Ox receptor1 antagonist prevents these effects. The study demonstrates the facilitatory effect of OxA on dopaminergic transmission. The authors propose that feeding elicits OxA release in the VTA by hypothalamic orexinergic neurons and that OxA, by stimulating Ox receptor1 expressed in VTA neurons, promotes DA release in terminal areas, such as the NAcS.

Food is a primary stimulus essential for the organism’s survival, since it assures the necessary caloric resources to pursue physiological activities. Unfortunately, in our contemporary societies, food can also acquire addictive-like properties promoting excessive consumption of highly palatable diets and increasing the risk of developing obesity and eating disorders. The potential capability of sugar to induce addiction-like behaviors has been well described and analyzed by Wiss et al. The authors illustrate their elegant animal model for sugar addiction that satisfies many of the criteria for “substance use disorder” reported in the *Diagnostic and Statistical Manual of Mental Disorders, Fifth Edition* (*DSM-5*). According to the model (15), rats develop a binge-like pattern of sugar intake and show behaviors similar to those observed in response to drugs of abuse, craving and withdrawal signs included. Interestingly, sugar withdrawal has been observed after sugar deprivation or naloxone injection. This is not surprising in view of the sucrose-elicited release of endogenous opioids. Furthermore, morphine or sugar withdrawal is accompanied by decreased DA and increased acetylcholine release in the nucleus accumbens (NAc). Moreover, while the NAcS dopaminergic response to palatable food habituates after repeated exposure, habituation does not develop in sugar-dependent animals, as observed in response to drugs of abuse. Other neurochemical similarities between rats dependent on drugs of abuse and sugar are reported, supporting the hypothesis that highly palatable foods “highjack” the neural systems that control responses to rewards, similar to what drugs of abuse do.

Like sugar, junk food diets can also modify several aspects of motivated behaviors such as food-seeking and consumption. This issue has been investigated by Kosheleff et al. who studied whether, in rats, 6 weeks of junk food exposure disrupted the ability to choose which food to pursue on the basis of expected value and availability predicted by conditioned cues. Long-term exposure to junk food impairs decision-making processes. The authors hypothesize that rats given extensive junk food exposure acquire a persistent expectation of having access to junk food, and this influences decision making about other food-seeking actions. They conclude that repeated junk food diet consumption induces long-lasting impairments in the behavioral control systems that regulate energy balance and nutritional diversity, which may contribute to overeating and obesity in humans. These conditions result in poor quality of life and low life expectancy, as they are associated to increased risk for developing several chronic diseases, including diabetes, cardiovascular diseases, and cancer. Unfortunately, valid therapeutic strategies are not yet available. Here, Maccioni et al. report the effect of Saikosaponin A (SSA), a major active component present in *Bupleurum falcatum* L., on the consumption of butter or chocolate cookies in a rat model of overeating. The authors previously demonstrated that SSA administration strongly reduces self-administration and reinstatement of seeking behavior for chocolate in rats (16). In the present study, SSA reduced consumption of highly palatable cookies at doses that do not induce motor incoordination or sedation. The authors suggest that the activation of 5-HT_2C_ receptors may underlie SSA effects, but specific studies should address this hypothesis.

The desire to decrease food intake in order to lose excess weight is quite common in our societies, and coffee is often used with this aim. However, clinical studies reported inconsistent effects of caffeine on appetite and calorie intake. In their study, Correa et al. analyze the effects of acute caffeine administration in mice on intake of highly palatable sweet food under different conditions. Caffeine administration under a schedule of intermittent access to highly palatable food that induces binge-like eating patterns increases intake. Under anxiogenic conditions, it decreases the amount of food consumed in 15 min, without modifying classical anxiety measures. Under effortful conditions, caffeine administration does not impair orientation toward food and increases speed, facilitating goal achievement. Moreover, it restores performance to normal effort levels when anergia and fatigue are induced by DA depletion. Effort-related motivated behaviors can be impaired in several diseases and caffeine may be a useful add-on treatment for motivational deficits in depression and other disorders.

Collectively, the articles presented in this *Frontiers* Research Topic provide a view of current theoretical and experimental work in the field and allow new insights into the mechanisms that underlie behavioral control of food intake and their possible dysregulation.

## Author Contributions

VB wrote the article. CG critically revised and approved the final version of the manuscript.

## Conflict of Interest Statement

The authors declare that the research was conducted in the absence of any commercial or financial relationships that could be construed as a potential conflict of interest.
